# 

**Published:** 1890-02

**Authors:** 


### Southern Medical Record

A Monthly Journal of Medicine and Surgery.

VOL. XX. ATLANTA, GA., FEBRUARY, 1890. NO. 2.

LIBRARY
Indexed
S. G. O.

EDITORS:

A. W. GRIGGS, M. D. FRANK O. STOCKTON, M. D.

WM. PERRIN NICOLSON, M D. DAN. H. HOWELL, M D., Bus. Mgr.

Entered at the Postoffice at Atlanta, Ga., as Second-Class Matter. Contents on Page 5

Gress & Sexton, Publishers, 47 S. Broad, Atlanta, Ga.

